# Laser Therapy for Vulvar Lichen Sclerosus, a Systematic Review

**DOI:** 10.3390/life13112146

**Published:** 2023-10-31

**Authors:** Ana Gil-Villalba, Angela Ayen-Rodriguez, Maria Jose Naranjo-Diaz, Ricardo Ruiz-Villaverde

**Affiliations:** Dermatology Department, Hospital Universitario San Cecilio, 18016 Granada, Spain

**Keywords:** lichen sclerosus, laser therapy, carbon dioxide laser, Neodymium:YAG laser, diode laser, systematic review

## Abstract

Lichen sclerosus (LS) is a chronic inflammatory disease that mainly affects the anogenital area, with a higher incidence in post-menopausal women. In the long term, it can lead to loss of vulvar architecture or progress to squamous cell carcinoma. The evidence-based treatment involves high-potency topical corticosteroids in long regimens. However, second-line treatments are not well-established, including laser therapy. This current study aims to assess the level of evidence supporting this therapy. We conducted a search for primary-level studies published before April 2023 through MEDLINE/PubMed, Embase, Web of Science, Scopus, and CENTRAL, with no restrictions on the publication language or date. The methodological quality and risk of bias of the included studies were evaluated using the updated Cochrane Collaboration’s tool for assessing risk of bias (RoB-2). Six studies (177 patients) met our eligibility criteria. Laser therapy was compared to topical corticosteroid treatment in five out of six studies. No significant histological differences were found, except for an increase in collagen production in the laser group. A greater reduction in itching, pain, and dyspareunia at 1 and 3 months of treatment in the laser group, as well as in the Skindex-29 at 6 months, was reported. Patient satisfaction was significantly higher among those who received laser therapy. Tolerability was excellent. No significant differences were observed in any of the previous aspects in the study compared to the placebo. In conclusion, there is not enough evidence to recommend laser therapy as a standalone treatment.

## 1. Introduction

Lichen sclerosus (LS) is a chronic inflammatory disease that mainly affects the anogenital area. It is more frequent in women, in a 3:1 or 10:1 ratio with respect to men, with a higher incidence in post-menopausal women. Incidence is estimated at 0.1–0.3%, but if we consider patients over 80 years of age, it amounts to 1.6%. The prevalence in private gynecological consultations is 1.7%, although these data are probably underestimated due to the lack of awareness of the disease, especially in younger women [1,2].

**Figure 1:**
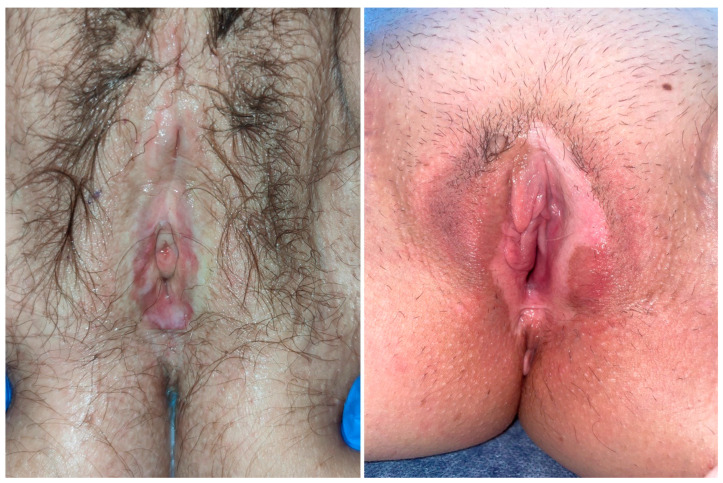
On the left, a white, atrophic plaque with central erythema and fissures in the genital area, accompanied by the fusion of the labia minora and the loss of the clitoris. On the right, an image displaying similar characteristics, with more pronounced features on the left labium minora.

Clinically, it is characterized by the formation of white, atrophic plaques, sometimes with erosions and fissures in the genital area. Long term, there is a loss of vulvar architecture with fusion of the labia minora, narrowing of the introitus, and burying of the clitoris, sometimes requiring surgical intervention (Figure 1). Pruritus, soreness, dyspareunia, and anal discomfort are common symptoms and have a significant impact on quality of life and sexual well-being [1,2,3,4]. Untreated LS is associated with a 4–6.7% risk of developing vulvar squamous cell carcinoma (SCC) [5,6]. The LS-mediated pathway leads to vulvar intraepithelial neoplasia (VIN), a differentiated type that leads to keratinizing SCC. It occurs in older women with untreated LS or lichen planus [7].

Diagnosis is fundamentally clinical, so a scoring system—clinical score system in vulvar lichen sclerosus (CSS VLS)—has been created, which includes typical signs and scores according to severity, with a rating >4 being diagnostic of LE with 90% certainty (Table 1). A biopsy is not necessary except in cases of non-response to treatment, suspected malignancy, extragenital involvement, or in young patients [3,4]. Pathologic findings usually include hyperkeratosis and epidermal atrophy with loss of rete ridges, and upper dermal changes with homogenization of collagen and a lichenoid inflammatory infiltrate. Early LS can be histologically misdiagnosed as eczema or “non-specific vulvitis,” and this should maintain our clinical suspicion of LS [7].

The etiology of LE is unknown. To date, the available evidence suggests two possible pathogenic mechanisms in individuals with a susceptible genetic background and exposure to certain triggers. Firstly, immune dysreactivity and chronic inflammation would play a key role in this disease. There is a high percentage of patients with other associated autoimmune diseases (thyroiditis, alopecia areata, vitiligo). In addition, an abnormal activation of the immune response mediated by Th1 lymphocytes has been found in affected tissues, and an overexpression of microRNA-155 (miR-155) would induce a suppression of regulatory T cells. On the other hand, increased fibroblast activity and abnormal collagen synthesis would assist in the progressive formation of hyalinized and sclerotic dermal tissue [8]. A genetic predisposition has been described, with a first-degree family history found in up to 12% of patients and related to an increased frequency of HLA-DQ7, –DQ8, –DQ9, and –DR12 in different cohorts; HLA-DR17 shows a negative association [1,7,8].

Possible triggers include chronic irritation and repeated trauma as part of the Koebner phenomenon [1]. Chronic scratching, previous surgeries or radiotherapy, urinary incontinence, multiparous condition, and high body mass index are associated with LE in older women. The role of hormones is controversial; there is a bimodal peak incidence in premenarchal and postmenopausal women, but the involvement of estrogens has not been demonstrated. Tissue estrogen receptors are found in equal proportion in healthy women and in women with LE, although with variants in their isoforms, which could explain the ineffectiveness of estrogen treatment. Other studies postulate that low levels of 5α-reductase could contribute to the onset of the disease so that anticonceptives with an antiandrogenic profile could influence early onset. In contrast, those based solely on progesterone would be protective. No infectious triggers have been related [1,2,3,4].

Treatment should focus on the control of inflammation and improvement of the sequelae (structural changes). First-line treatment with a super-potent topical corticosteroid (TC) and a clobetasol propionate (CP) is considered to be evidence-based therapy, used from 6 to 12 weeks, and then followed by a maintenance regimen of 2–3 times per week [1,2,3,4,9,10,11,12,13,14,15]. A lack of response to corticosteroid therapy should lead us to investigate concomitant causes such as contact dermatitis. Correct treatment with CT has been shown to reduce the risk of VSCC. A prospective longitudinal cohort study conducted on 507 women, during a mean 4.7-year follow-up, demonstrate that patients who complied with corticosteroid treatment did not develop VSCC [16]. Chin et al. [17] published a lower 5-year recurrence rate of vSCC or VIN in patients who were treated with topical corticosteroids versus those who were not (27% versus 44% to 47%).

Adjuvant treatments could help reduce the use of corticosteroids and also help symptom control. Second-line treatments are topical calcineurin inhibitors, tacrolimus 0.1% ointment, or pimecrolimus 1% cream once or twice a day for 1–2 months. Randomized trials demonstrate their effectiveness and safety. Nevertheless, CP seems to be more effective and has better histologic efficacy, and it should remain as the first-line therapy [9,10]. Topical alternatives as a third-line therapy include retinoids (tretinoin 0.025% in cream and cis-retinoic acid 0.5% in ointment) that regulate the process of keratinization and sclerosis. Caution should be taken with the secondary discomfort to the retinization process [11]. Systemic treatments that have been reported to be beneficial in small numbers of patients include oral acitretin (20–30 mg/day), cyclosporine, methotrexate (with previous corticosteroid pulses), and adalimumab [12,13,14,15]. As for light-based therapies, photodynamic therapy (PDT) using topical 5-aminolevulinic acid can be considered a valid option for incoercible pruritus in LS when conventional therapies have failed. Nonetheless, changes in histopathologic inflammation were inconsistent, so additional research is needed to determine the efficacy of this treatment modality [15].

Laser therapy is an emerging therapy, although evidence is weak and long-term data supporting its effectiveness are lacking [2]. The most studied is the fractional carbon dioxide (CO_2_) laser (FxCO_2_). This is a laser that operates at a wavelength of 10,600 nm and targets intracellular water, causing selective heating and vaporization of the tissue, resulting in superficial microabrasion. It uses a pulsed beam to avoid overheating, and the fractionated mode delivers energy in columns of alternating treated and untreated tissue areas. In this way, the burn is more controlled, and the recovery time is shorter. At the dermal level, we are interested in the fragmentation of the collagen matrix and the promotion of new collagen formation and neovascularization, stimulating tissue repair and remodeling. Some have compared this effect to wound healing [18]. This remodeling effect is the basis for scar treatment or facial rejuvenation with FxCO_2_ [19,20]. Despite good results in improving symptoms, clinical signs, and quality-of-life scales, some authors conclude that it is not superior to TC and is not sufficiently effective in monotherapy. This evidence is mostly based on small non-controlled studies and case series; there is a notable absence of high-quality trials that confirm its efficacy. Other types of laser modalities explored are fractional erbium-doped yttrium aluminum garnet (Er:YAG) lasers, non-fractionated ablative CO_2_ lasers, and diode lasers [2,18].

With the aim of gathering evidence about this therapy in vulvar LS, we have performed the present systematic review.

## 2. Materials and Methods

The current systematic review was conducted following the criteria established by the Cochrane Collaboration and strictly adhered to the reporting guidelines outlined in the Preferred Reporting Items for Systematic Reviews and Meta-Analyses (PRISMA) [21]. With the aim of reducing potential bias, a protocol detailing the methodology employed in this systematic review was formulated and registered in the PROSPERO international prospective register of systematic reviews before the study commenced.

### 2.1. Search Strategy

An extensive search was conducted across multiple electronic databases, including MEDLINE (via PubMed), Embase, Scopus, Web of Science, and the Cochrane Central Register of Controlled Trials (CENTRAL). The search strategy was developed by combining controlled vocabulary terms (Thesaurus terms) with free-text terms (please refer to Supplementary File S1). There were no restrictions placed on the publication year or language. Additionally, a manual search of the reference lists of all selected studies was performed to identify any additional relevant publications. This comprehensive search was carried out in April 2023. To manage the retrieved records, a bibliographic reference management software was employed (Mendeley version 1.19.8, Elsevier, Amsterdam, The Netherlands).

### 2.2. Selection Criteria

The following inclusion criteria were taken into account:

- Clinical trials that evaluated laser treatment in LS compared to a control group;
- Studies focused on women with proven genital LS;
- Studies reporting the clinical response to this therapy, whether histological, clinical, or quality-of-life scales.

The exclusion criteria were as follows:

- Other types of studies apart from clinical trials, including other systematic reviews or meta-analyses;
- In vitro or in vivo animal trials;
- Extragenital forms of LS and male patients.

### 2.3. Study Selection and Data Extraction

Studies were chosen based on predetermined inclusion and exclusion criteria by two blinded reviewers (AGV and RRV). Any discrepancies were resolved by a third impartial individual (AAR). The selection process for the studies occurred in two distinct phases. Initially, it involved assessing the titles and abstracts of the articles. Subsequently, a comprehensive reading of the articles selected in the previous phase was conducted. Data extraction from the chosen articles was independently performed by two authors (AGV and RRV) using a standardized data extraction template. The information collected included the first author, publication date, study design, number of enrolled patients, sex of patients, previous biopsy proving LS, details of the intervention and comparison, laser modality, treatment protocol, and duration of follow-up. Outcomes analyzed were histological changes, clinical assessment tools for symptom evaluation, involvement in quality of life, and degree of improvement from the patient’s and clinician’s point of view, as well as clinical images.

### 2.4. Risk of Bias Assessment

The methodological quality of the primary-level studies was rigorously evaluated using the Cochrane Collaboration’s RoB-2 tool for evaluating the risk of bias [22]. Various potential biases were examined and categorized into five specific domains, which encompassed: (1) The randomization process; (2) Deviations from intended interventions; (3) Missing outcome data; (4) Measurement of the outcome; and (5) Selection of the reported result. Each domain was assessed independently in each primary-level study, with specific details and potential biases noted. A sixth and final domain evaluated the overall risk of bias, taking into account the findings from the previous five domains. To visualize the risk of bias, risk of bias plots were generated using the Cochrane robvis web app.

### 2.5. Synthesis of Results

Data were analyzed using narrative synthesis methods, which enabled the examination of both qualitative and quantitative data derived from primary studies. Data are presented in tables, organized by the year of publication, along with their most significant methodological, scales evaluation, and results. Meta-analysis was not performed because of the expected methodological and clinical heterogeneity between studies, mainly attributed to differences regarding the type of intervention, the control group, and the variables used to assess outcomes. As a consequence, meta-analytic techniques such as the computation of pooled estimates, evaluation of statistical heterogeneity, and forest plot construction were not carried out.

## 3. Results

### 3.1. Study Selection

**Figure 2:**
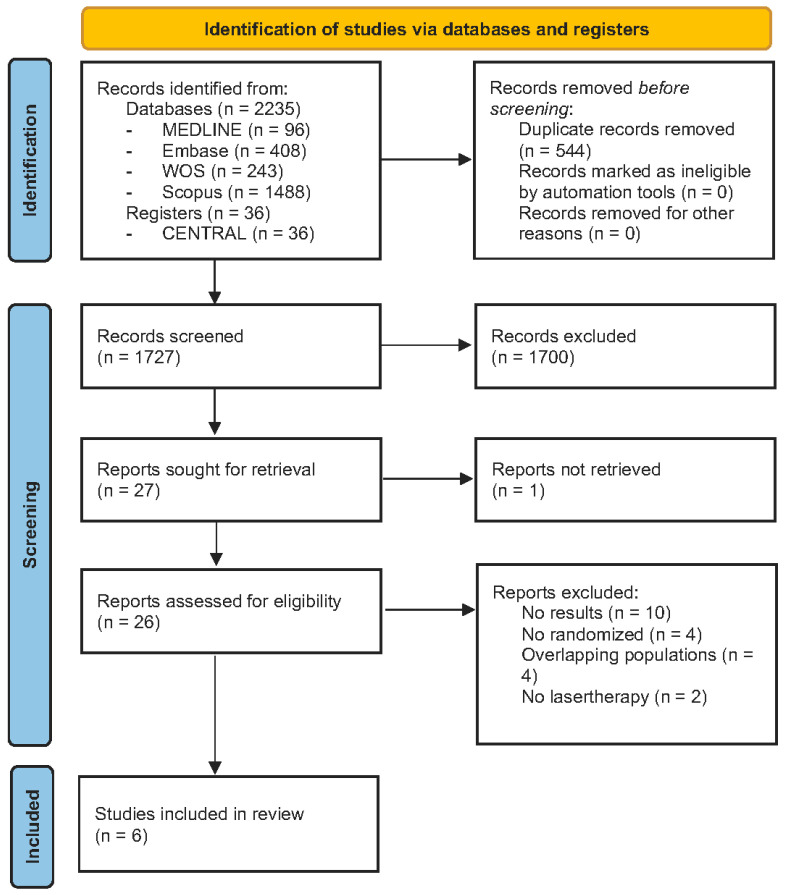
Flow diagram of the selection process of primary-level studies.

Figure 2 illustrates the flow diagram of the process of identifying and selecting studies. The search strategy across databases and registers retrieved a total of 2271 studies: 96 from PubMed/MEDLINE, 408 from Embase, 243 from Web of Science, 1488 from Scopus, and, finally, 36 clinical trials from CENTRAL. After duplicates were removed, a total of 1727 was remaining for screening by title and abstract analysis. A total of 27 studies were assessed by full-text review, of which 21 did not meet the eligibility criteria and were consequently excluded. Only six studies met the inclusion criteria, and they were included in the final sample for data extraction and analysis [23,24,25,26,27,28].

### 3.2. Study Characteristics

Of the six studies analyzed, all were controlled clinical trials published between 2017 and 2021 in different media, two of them in journals and four in conference abstracts. The total sample consisted of 177 female patients with biopsy-confirmed LS diagnosis. They were divided into a laser treatment group and a control group.

Table 2 summarizes the characteristics of the selected studies. Three different types of lasers were used: fractional CO_2_ laser, Nd:YAG, and Diode Laser with considerable variability in the number of sessions and the time between them. Two studies employed diode lasers weekly for 8 weeks (w/λ = 660 nm, P = 100 mW, I = 510 mW/cm^2^, E = 4 J, R = 20 J/cm^2^, T = 40 s). They were a continuation of each other with an analysis of different parameters. Another study examined the effect of three sessions of the Nd:YAG laser every 2 weeks (9-mm spot size, Piano pulse (5 s), R = 90 J/cm^2^) concomitantly with topical Betamethasone pre- and post-treatment. The fractional CO_2_ laser was used in three studies. Two of them are also a continuation of each other by analyzing long-term data with crossover. It is important to note that they do not overlap in the publication of results. They used a vulvovaginal CO_2_ laser every 4–6 weeks for a total of three sessions (baseline session P = 26 W, dwell time 800 µs, DOT spacing at 800 µm, the two additional ones P = 30 W, dwell time 1000 µs, spacing 1000 µm). The other one does not specify parameters, but it is the only one that used a control group with a placebo, which is a very small amount of laser energy that produces visible spots and creates smoke and odor from the vaporization of the skin without interfering with the pathological process. The control group in the rest of the clinical trials used topical corticotherapy in a descending pattern for a period of 4-to-8 weeks.

### 3.3. Patients Features

Patients were all females, with a total N of 177, for which demographic variables are not detailed in most studies because they are abstracts. The majority were post-menopausal women with a mean age of 59 in three studies; most were Caucasian, while only one black patient was detailed.

### 3.4. Risk of Bias Analysis

**Figure 3:**
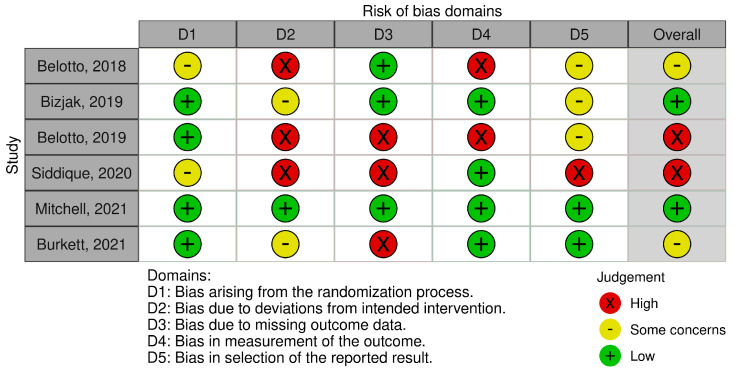
Quality plot showing risk of bias of primary-level studies, using the Cochrane Collaboration’s tool for assessing risk of bias [23,24,25,26,27,28].

**Figure 4:**
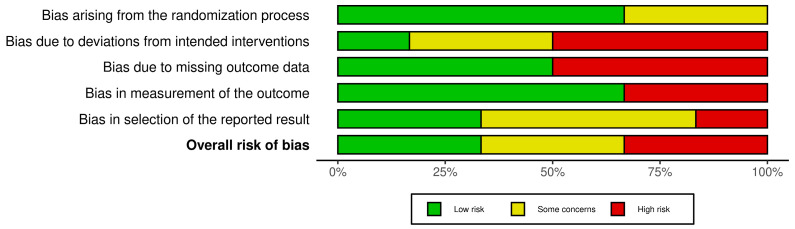
Summarize plot showing risk of bias by domain, expressed as percentages.

Figure 3 and Figure 4 show a summary of the risk of bias and the resulting graph after evaluating the studies included. Thirty-three percent of the studies were considered to have a high risk of bias, 33% had some concerns, and only two were considered to have a low risk. As mentioned above, all of the studies were randomized trials, in which the order of randomization is concealed. However, the patient was aware of the assigned treatment. Thus, 66% of the articles scored a low risk for randomization bias, while 33% registered some concerns. Regarding bias due to deviations from the intended intervention, only one article was determined to be of low risk, and it was the only one compared with the placebo. The rest were high risk (50%) or some concerns (33%). The bias due to missing outcome data was equally distributed, with a 1:1 ratio of high and low risk. Bias in outcome measures was low risk in almost 66% of trials, while only two (33%) were high risk. Finally, bias in the selection of the reported result involved low risk or some concerns in 83% of the studies.

### 3.5. Clinical and Histopathological Outcomes

A summary of the results published in the articles is provided in Table 2. Different evaluation methods were used to measure treatment response. In terms of symptom improvement, some studies found significant differences in the use of lasers. Bizjak et al. [24] found a statistically significant reduction in all symptoms (VAS burning, itching, pain, and dyspareunia) at 1- and 3- months follow-up in the laser group compared to baseline parameters and compared to corticosteroid therapy. They also described improvement in the quality of sex life only in the laser group, although with problems recurring at 6 months. There were no statistically significant differences in the clinical changes as measured by photos. Burkett et al. [26] demonstrated a more significant improvement in the Skindex-29 score in the laser-treated group at 6 months, with an effect size of 11 points (*p* = 0.007). In addition, the change in mean scores for the VSQ [−3.92 (SD 4.12) versus −0.58 (5.11), *p* = 0.014] and VHI [1.92 (SD 4.34) versus 0.43 (3.62), *p* = 0.046] scales were significantly better in the laser group at the 6-month assessment. Mean VAS scores were comparable between the two groups. When stratified according to previous steroid use, the notable change in Skindex-29 score was only observed in the group that had previously been treated with steroids. This study is continued in another publication by Sidiqque et al. [28]. After 6 months of follow-up, the patients who had not responded changed groups and were evaluated for a further 6 months. The final results at 12 months showed the lowest scores in the Laser-Only group (LOG) and the highest in the Steroid-Only group (SOG). The LOG exhibited similar scores to the crossover groups (Steroid to Laser and Laser to Steroid) at 12 months. The Steroid-to-Laser group had a greater decrease in the overall score with the addition of the laser (−10.23, 17.53 SD). The validated CSS –VLS was used in one of the studies with no statistically significant difference found between the laser group and the placebo group (minimal energy laser) [27].

In terms of histopathology, Belotto et al. [23] found that the laser diode group and corticoid group were similar in their post-treatment results in terms of the reduction of the inflammatory process and improvement of the epithelial aspect. They assess severity according to Robboy’s classification VLS1 (initial or stabilized) and VLS2 (old). Pre-treatment VLS 2 was 86.7% in both groups, then in post-treatment, it persists to 33.3% and 26.7%, respectively. This same working group in another conference abstracts [25] studied in pre- and post-treatment biopsies the collagen and inflammatory infiltrate. Laser treatment was superior to standard treatment in collagen stimulation, while corticosteroids maintained vulvar skin thinning. The corticoid does not favor collagen improvement, maintaining vulvar skin thinning. Bizjak et al. [24] showed that sclerosis was greatly reduced with the laser (−0.67 mm; 95% CI −0.99 to −0.34 mm; *p* = 0.009), whereas other histological variables, such as the thickness of the epidermis and the degree of inflammation, did not differ compared to topical corticosteroid group. Mitchell et al. [27] found no significant differences between the laser and placebo groups.

Laser treatment was well-tolerated; someone described a minimal treatment discomfort as a sensation of warmth, which improved to the point of disappearance as the number of sessions increased [24]. The different studies do not report any adverse effects; only one study reports minor burning and blistering. In one case, this was treated with lasers and a herpetic reactivation in the corticosteroid group [26].

Regarding patient global satisfaction, half of the included studies assessed this area (3/6) [24,26,27] using ordinal scales ranging from “very unsatisfied” to “very satisfied”. Some studies found a higher degree of satisfaction in the group of patients undergoing laser treatment compared to those treated with steroids (81% were “satisfied or very satisfied” versus 41%, *p* = 0.01). It also increased the degree of satisfaction when they were changed to the laser group (Steroid-to-Laser group (52%) versus the Laser-to-Steroid group (22%), *p* = 0.001) [27]. In addition, Bizjak et al. found higher satisfaction in the laser treatment group, with 100% of patients being “very satisfied” compared to only 2% of the CT patients (χ² = 36.4; *p* < 0.001) [24].

## 4. Discussion

A systematic review of laser treatment in LS allows for a comparison of the evidence published to date and comparison with established therapies such as TC. It evaluates the safety of the treatment and identifies additional benefits such as satisfaction, tolerability, and reduced side effects compared to other therapeutic options.

### 4.1. Limitations of the Studies

Among the published articles, there are few randomized clinical trials, and they carry a high risk of bias. First, we lack sufficient information on the methodology used in the studies, probably because they are conference abstracts with a character limitation. In addition, the trials are not blind because of the difficulty of masking a physical treatment such as laser; only one of them uses placebo, a low-energy laser with sufficient power to mask the trial to the clinic and the patient. The remaining articles use high-potency TC as a control group. However, the period of use is relatively short compared to that recommended in the guidelines, which advise continuous use for 6-to-12 weeks followed by an indefinite maintenance regimen. For example, Bizjak et al. [24] only use them for 4 weeks at decreasing doses, which in daily clinical practice is insufficient for LS.

Another limitation lies in the restricted patient information available. There is no clarity regarding the duration of the disease, prior treatment history, whether they were corticosteroid-naïve, or if they had been unresponsive to multiple treatments, all of which are adverse prognostic factors that considerably influence clinical responses. Neither do they demonstrate long-term follow-up (maximum follow-up period of 12 months), nor are maintenance regimens combining these therapies proposed. If we look back at Figure 3 of the risk of bias domains, the most frequent limitations or biases, in ≥50% of the studies, are due to: (1) The patients were aware of the intervention, which would be solved with blind clinical trials controlled with placebo, laser low energy; (2) A lack of information, due to the publication of incomplete results without patient or methodological details and unjustified losses to follow-up. This would be corrected by having all the data from the trials and not just conference abstracts.

Regarding the assessment scales for LS and its response to treatment, there is no consensus among studies that would allow for collective analysis, neither on a clinical, psychological, nor histological level. There is a validated tool for assessing the severity of CSS VLS [3], which is relatively unknown and was only used in one of the studies. The extent of its use could help us compare results between studies. On the other hand, photographic follow-up, although potentially more objective, has not shown significant differences in any study, which may be attributed to the fact that symptoms and signs do not have an exact correlation and improvement does not occur simultaneously. In a published case series involving 244 symptomatic women with LS treated with TC, 66% of patients experienced complete relief of symptoms, while only 23% of patients achieved complete resolution of clinical signs, including the return to normal color and texture [29].

### 4.2. Outstanding Findings

The role of FxCO_2_ for the genitourinary syndrome of menopause (GSM) is controversial. The low quality of studies and lack of information on efficacy and safety have led to the discouragement of FxCO_2_ as a treatment. Results are mixed, while some randomized control trials argue for its superiority over placebo or in combination with estriol, showing significant improvements in symptoms and histology [30,31]. Other trials find no symptomatic or histological differences [32]. When it concerns LS, the results cannot be extrapolated. In most cases of GSM, there is a mild inflammatory infiltrate. In contrast, LS triggers an intense local inflammatory response leading to lichenification and sclerosis of the genital area. TC helps combat the inflammatory response, but they are often insufficient to control symptoms. Laser therapy has been shown to reduce cutaneous sclerosis and increase local collagen synthesis, so its simultaneous use with TC would help promote collagenogenesis and mitigate the symptoms of this pathology [23,25,26].

The advantages of this combination are suggested by the results of the studies analyzed. Firstly, Burkett [26] highlighted that after stratification by previous steroid use, the group that showed the most changes in Skindex-29 was the one that had previously been treated with corticosteroids. Then, Siddique [27], with his crossover RCT, showed that patients who were switched to another group because they had not responded to monotherapy had better outcomes. It should be noted that these studies were not originally intended to address the efficacy of combination treatments, so protocols for such purposes could be considered in future studies.

Another advantage is related to patient satisfaction, which has been observed to increase in the laser-treated group. In fact, one of the studies found difficulties in the adherence of the control group due to the low motivation to use TC. Many of the patients entering the trials come from long periods of treatment with corticosteroids with little improvement or were seeking a definitive solution to LS. It is well-established that treatment adherence is crucial for enhancing the quality of life in these patients, and the dropout rate is significantly higher when no response is obtained within a few weeks of initiation [29,33,34]. One study identified low treatment adherence and disease control as primary risk factors for lower quality-of-life (QOL) scores. In the group with the poorest QOL (pQOL), there were significantly higher proportions of patients with partial adherence (*p* = 0.006), suboptimal disease control (*p* < 0.001), and scar progression (*p* = 0.024) [34]. Therefore, we emphasize again that it seems important to associate other therapies with the gold standard to maintain motivation levels and the perception of improvement.

In summary, to date, there is no sufficient evidence to recommend laser therapy as monotherapy. The main findings from which this statement is derived are as follows: There are few published clinical trials, most of them have a high risk of bias, and they chose control therapy that is not daily clinical practice. Furthermore, the efficacy of the use of laser in combination with CT has already been evaluated in some of the studies presented here. Consequently, the recommendations made in previous paragraphs should be evaluated for future studies, with the aim of obtaining more robust evidence on the use of laser therapy, alone, or in combination with CT.

## 5. Conclusions

Despite the disparate results and lack of significant differences between TC therapy and laser treatment in many of the studies, we can draw interesting conclusions from them. (1) Laser treatment in combination with TC can help mitigate dermal thinning and skin atrophy by promoting the remodeling of sclerosed tissue and collagenogenesis. (2) Laser treatment increases patient satisfaction, which results in greater therapeutic adherence and clinical improvement measured through different symptom indexes. However, more studies are needed to support this evidence, including larger blinded randomized controlled studies.

## Figures and Tables

**Table 1:** Clinical score system in vulvar lichen sclerosus (CSS VLS) [1,3,4].

	Grade 1 (Moderate Changes)	Grade 2 (Severe Changes)
Erosions	1–2 small erosions, almost not macroscopically visible	Macroscopically visible and/or more than 2 or confluent lesions.
Hyperkertatosis	Affecting the vulva and perineum up to 10%	Affecting the vulva and perineum more than 10%
Fissures	Rhagades affecting the posterior introitus	Generalized vulvar rhagades
Agglutination	Partially affecting preputium clitoridis and labia minora	Complete agglutination of both
Stenosis	Narrowing of the introitus that could still be passed by two fingers	A narrowing that could be passed by less than two fingers
Atrophy	Shrinkage of labia minora and clitoris	A narrowing that could be passed by less than two fingers

**Table 2:** Study characteristics and evaluated outcomes.

Study	Design	n	Intervention	Scales/Parameters	Results	Follow Up	Conflicts of Interest
Belotto R., 2018 [23]	RCT	30 ♀	LG: CW Diodo Laser weekly for 8 weeks (w/λ = 660 nm, P = 100 mW, I = 510 mW/cm^2^, E = 4 J, R = 20 J/cm^2^, T = 40 s) CG: 0.05% clobetasol propionate ointment every 24 h for 4 weeks, then every 48 h for 4 weeks.	Histological changes (VLS1 y VLS2)	Both groups show inflammatory reduction and epithelial aspect improvement post-treatment. LG 86.7% vs. 33.3% VSL2 pre- and post-treatment; CG 86.7% vs. 26.7%.	8 weeks, Control biopsy 90 days after	No stated
Bizjak Ogrinc U., 2019 [24]	RCT	40 ♀	LG: Nd:YAG laser every 14 days, 3 sessions (SP Dynamis, Fotona, Slovenia, using R33 non-contact handpiece, 9-mm spot size, Piano pulse (5 s), R = 90 J/cm^2^) Diprosone^®^ 1 week pre-treatment + Diprogenta^®^ 2 days post-treatment CG: topical betamethasone (Diprosone^®^) for 4 weeks with decreasing dose	VAS (burning, itching, pain) Tolerability VAS Histological Clinical photographs Patient satisfaction (0–3)	At 1–3-month follow-up, patients in the laser group had significantly greater improvement in LS symptoms. At the 6-month follow-up, this improvement was still significant. Patient satisfaction was significantly higher in the LG than in the CG 3 months after treatment (*p* < 0.001). The correct order of photographs was assigned significantly more often in the LG (15/20 vs. 4/11 in CG; *p* = 0.035). The change in histopathology scale score between arms was not statistically significant. Laser treatment discomfort was on average 1.5/10 on the VAS.	1, 3, 6 months	No stated
Belotto R., 2019 [25]	RCT	12 ♀	LG: Diode Laser, l = 660, P = 100 mW, SD = 510 mW/cm^2^, E = 4 J, DE = 20 J/cm^2^, T = 40 s. once weekly for 8 weeks. GC applied clobetasol propionate 0.05% ointment, daily for 4 weeks, after alternated days for 4 weeks.	VAS Histological Thermography	CG inflammatory infiltration decreased post-treatment. However, the collagenous hyalinization still remained. PBMG inflammatory infiltration decreases and collagen matrix remodeling was observed post-treatment. The post-treatment pruritus showed a significant diminution in both groups. The thermography local heat intensity decreases in the post-treatments; in the PBM group. it was more relevant.	No stated	No stated
Burkett L., 2021 [26]	RCT	52 ♀	LG: Fractional CO_2_ laser (Vulvovaginal SmartXide2–V2-LR laser system fractionated CO_2_ laser) 3 sessions. Baseline treatment (P = 26 W, dwell time 800 µs, DOT spacing at 800 µm), 2 additional sessions 4–6 weeks apart (P = 30 W, dwell time 1000 µs, spacing 1000 µm) CG: Topical clobetasol propionate: nightly 1 month, 3 t/w 2 months, then as needed.	Skindex-29 Patient satisfaction: PGI-S PGI-I VAS VSQ VHI	Fifty-two participants, 27 pts each group, one dropout in steroid group. At 6 months greater improvement laser arm: Skindex-29 (11-point effect size, *p* = 0.007), mean VSQ [−3.92 (SD 4.12) vs. −0.58 (5.11), *p* = 0.014] and VHI [1.92 (SD 4.34) vs. 0.43 (3.62), *p* = 0.046]. Mean subjective and objective VAS were similar between groups. Eighty-nine percent of laser patients rated their symptoms as being “better or much better” on PGI-I compared to 62% of steroid patients, *p* = 0.07. Eighty-one percent of laser patients were “satisfied or very satisfied” on PGI-S compared to 41% of steroid, *p* = 0.01. After stratification for previous steroid use, the significant change of Skindex-29 score was only seen in the previously exposed group.	6 months	No conflicts of interest
Mitchell L., 2021 [28]	RCT	40 ♀	Active arm: Fractionated CO_2_ group, 5 sessions in a 24-week period. Sham laser group, 5 sessions (FxCO_2_ with very minimal laser energy emitted).	CSS patient + investigator’s impressions. Biopsy (0–6 scale) Photos	Twenty patients in each group, three women excluded for not having control biopsy. Histopathology scale score: 0.2 reduction (improvement) in active group (95% CI −1.1, 0.80, *p* = 0.74), front 0.1 increase in sham group (95% CI −0.90, 1.0, *p* = 0.91). Difference not statistically significant in the ITT analysis (−0.2; 95% CI −1.14, 1.06, *p* = 0.76). CCS: 7.10-point reduction in active arm (95% CI −13.2, −1.1, *p* = 0.02); 4.80-point reduction in sham group (95% CI −9.50, −0.20, *p* = 0.04). Difference not statistically significant *p* = 0.60. In the physicians’ point CSS, there was a 0.70 increase (worsening) in the active (95% CI −0.80, 2.1, *p* = 0.36) and a 0.30 reduction in sham group (95% CI −1.7,1.2, *p* = 0.70).	24-week treatment period + 8-week follow-up	Additional funding for this study was supplied by El.En Group, Florence, Italy, the manufacturer of the laser used in this study. In addition, El.En Group supplied the laser used in the study.
Siddique M., 2020 [27]	RCT + crossed over	55 ♀	Continuation of the previous study. After 6 moths, the patients still symptomatic crossed over: Steroid to Laser group (StLG), Laser to Steroid groups (LtSG)	Skindex-29 Patient satisfaction PGI-S PGI-I VAS VSQ VHI	Fifty-five participants, 48 had 12-month follow-up, 7 losses. Of the 48, 21 crossed over (11 from steroid to laser, 10 from laser to steroid). At 6 months the greatest difference in Skindex-29 overall score was in the LG (−23.2, 19.0 SD). At 12 months, the lowest scores were in LG and highest in the CG. LG had similar scores to the crossover groups at 12 months. The StLG had a greater decrease in overall score with laser (−10.23, 17.53 SD) versus steroid (−4.38, 5.65 SD). The LtSG, with laser (−10.29, 12.18 SD) versus steroid (−1.52, 10.62 SD). There was a greater increase in patients who were “satisfied or very satisfied” on PGI-S in the StLG (52%) versus the LtSG (22%), *p* = 0.001.	6, 12 months	R. E. Gutman: Boston Scientific: Consultant and Grant/Research Support, Up To Date: Royalty, Johnson & Johnson: Expert Witness Sling Class Action; A. Park: Allergan: Speakers’ Bureau; C.B. Iglesia: Foundation for Female health Awareness made payable to MedStar Health Research Institute: Grant/Research Support.

RCT: randomized controlled trial; CG: control group; LG: laser group; VAS: Visual analog scale; VSQ: vulvovaginal symptom questionnaire; VHI: Vaginal Health Index; PGI-S: Patient Global Impressions scale—Severity; PGI-I: Improvement. CSS: Clinical Scoring System for Vulvar Lichen Sclerosus, Robboy’s classification VLS1 (initial or stabilized), VLS2 (old). PBMG: Photobiomodulation group.

## Data Availability

The data that supports the findings of this study are available in the Supplementary Material of this article.

## Supplementary Materials

The following supporting information can be downloaded at: https://www.mdpi.com/article/10.3390/life13112146/s1, Supplementary File S1. Search strategy.
