# Supplementary material for: Laser Therapy for Vulvar Lichen Sclerosus, a Systematic Review
Source: Life (Basel). 2023 Oct 31;13(11):2146. doi: 10.3390/life13112146 (PMC10672171; doi:10.3390/life13112146)
Supplement: Supplementary file 1 [file life-13-02146-s001.zip › life-2664274-supplementary.pdf]

Supplementary File S1. Search strategy.

**Database**	**Query/Search Strategy**	**Items founds/Results**	**Search time limits**
MEDLINE	(“Lichen Sclerosus et Atrophicus”[MeSH Terms] OR “Vulvar Lichen Sclerosus”[MeSH Terms] OR ((“lichen”[all] OR sclero*[all]) AND (vulv*[all] or genital*[all]) NOT (“male”[all] OR “balanitis”[all]))) AND (“Laser Therapy”[MeSH Terms] OR laser*[all])	96	January, 2023
Embase	Embase: (('lichen sclerosus et atrophicus'/exp) OR ('lichen sclerosus') OR ('vulva kraurosis'/exp) OR (('lichen' OR 'sclero*') AND ('vulv*' OR 'genital*'))) AND ('laser therapy'/exp OR 'laser'/exp OR 'laser*')	408	January, 2023
CENTRAL	(“lichen sclero*” OR “Lichen Sclerosus et Atrophicus” OR “Vulvar Lichen Sclerosus” OR ((lichen OR sclero*) AND (vulv* OR genital*))) AND (laser* OR “laser therapy”)	36	January, 2023
Web of Science	TS=((lichen sclerosus) OR ((lichen OR sclero*) AND (vulv* OR genital*))) AND TS=(laser*)	243	January, 2023
Scopus	ALL (((lichen OR sclerosus) AND (vulv* OR genital*)) AND (laser*))	1,488	January, 2023
Total			2,271
